# Damp Cellars

**Published:** 1887-10

**Authors:** 


### DAMP CELLARS.

The importance of having dry cellars, cannot be too strongly urged upon the people. We recently visited an afflicted family at Phœnix Park, near Pottsville, Pa., where five members of the family were ill with a typhoid disease, and two had died, making seven cases in all. We made a very thorough examination of this house, had the drinking water analysed, and were forced by exclusion to the conclusion that the sickness in this case was caused by a damp cellar. A stream from a worked out mine, kept the locality marshy, and the cellar wet. To obviate this a drain had been run from the cellar to a neighboring creek. This drain had been stopped, and some inches of water had accumulated in the cellar. Had this family known that dynamite was in the cellar, they would not have slept easily until it was removed, but with this insidious foe to life and health, they ate and slept contentedly, until the favorite child, a boy of eleven, was taken ill and died. Then, suspecting the damp cellar, the drain was cleaned out, but it was too late, the mischief was done, the family was infected, and all of the children had the disease. As I looked at the bereaved and saddened mother, I could but pity her want of knowledge, that had brought such affliction. The doctor could not cure, but the parents could have prevented. Do not live in a damp cellar an hour.—*Annals of Hygiene.*

